# A critical appraisal of qualitative research exploring the lived experience following total laryngectomy

**DOI:** 10.1097/MOO.0000000000001109

**Published:** 2026-01-24

**Authors:** Laura-Jayne Watson, Lisa Houghton

**Affiliations:** a University of Liverpool/South Tyneside and Sunderland NHS Foundation Trust; b Speech & Language Therapy Department, University Hospital Aintree, Liverpool University Hospital Foundation Trust, Liverpool, UK

**Keywords:** laryngectomy, lived experience, qualitative

## Abstract

**Purpose of review:**

Laryngectomy is a life-changing operation; however, little attention has been paid to exploring peoples lived experiences. Understanding these experiences is critical, particularly in relation to the psychosocial, communicative, and rehabilitative challenges that follow surgery.

**Recent findings:**

Critical appraisal and author discussion of eleven articles generated three inter-related themes:

(1) Relationships: people with a laryngectomy and their families
(2) Social roles after laryngectomy
(3) Functional changes and preparedness for life after laryngectomy

**Summary:**

Critical appraisal of the literature has provided a unique insight into the lived experience of people after laryngectomy. This literature review advocates the importance of transitioning long-term laryngectomy care away from a sole medical model to one that incorporates a biopsychosocial model of care. It would be beneficial for future work to explore a wider demographic of people with a laryngectomy. For example, a more diverse range of experiences across age, gender, ethnicity and socio-economic status. This would ensure that the needs of local populations are met within their local communities, consistent with principles of accessibility, equity and person-centred care.

## INTRODUCTION

Laryngectomy is a life-changing operation resulting in long-term functional changes to communication, appearance, breathing and swallowing [1,2]. There is a wealth of evidence in pre- and postoperative surgical management of laryngectomy [3–6]; however less attention has been paid to exploring peoples lived experience after laryngectomy. It is paramount to understand this given the changing landscape of healthcare service provision, particularly the emphasis on supporting people to get seamless, integrated care in the right place and at the right time to support their outcomes [7]. 

**Box 1:**
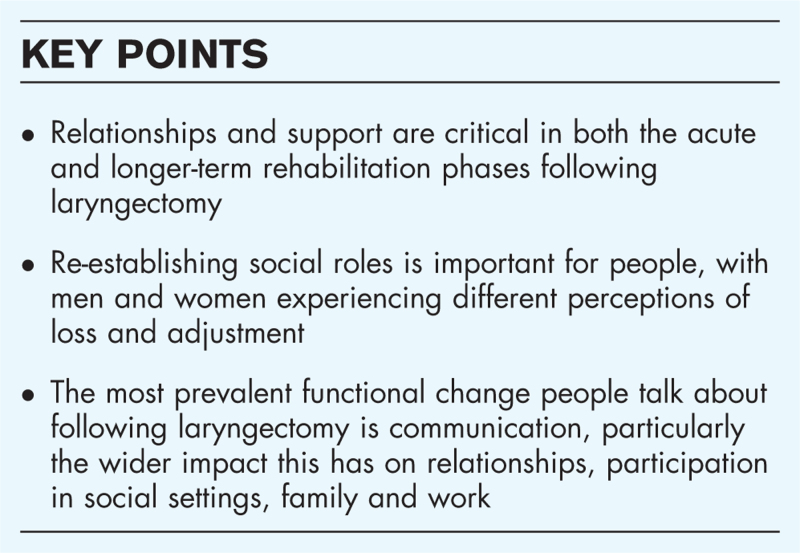
no caption available

This review aims to:

(1) Explore available evidence exploring lived experience after laryngectomy, including family members experiences
(2) Identify potential clinical implications and future areas for clinical research

## REVIEW

A literature search was conducted using Scopus, Medline, Web of Science, Ebsco and Science Direct in February–April 2024. The search was repeated in January 2025 and May 2025. Key words in the search terms included ‘laryngectomy’, ‘lived experience’ and ‘qualitative’. MeSH terms were exploded and Boolean operators (‘AND’, ‘OR’) and truncation used. The authors limited the literature search to any type of publication (article, book chapter) published within the last fifteen years. Non-English references were excluded.

Two hundred forty-seven publications were retrieved and potentially eligible for review. One hundred eighty-two of these were excluded (focussed on quantitative methods only, focus not on lived experience, for example, quality of life in tracheoesophageal voice users), leaving 65 publications. Forty-three duplicates were removed, and the remaining abstracts were screened for inclusion using an agreed definition of lived experience: *“personal knowledge about the world gained through direct, first-hand involvement in everyday events rather than through representations constructed by other people. It may also refer to knowledge of people gained from direct face-to-face interaction rather than through a technological medium.”*[8]. References from the identified publications were also hand searched.

**FIGURE 1:**
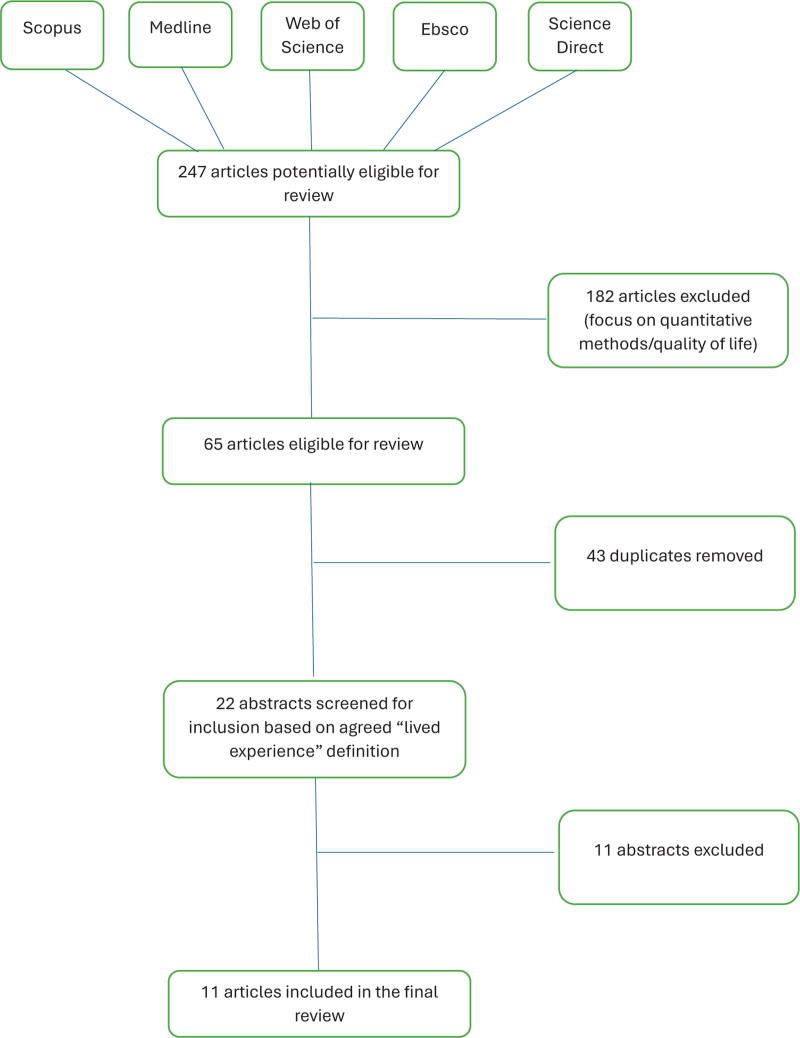
Review process and selection stages.

In total, 11 publications, all journal articles, were included in the final review which were all retrieved from the original literature search. No additional articles were included from the hand search (Fig. 1).

The final 11 articles were reviewed independently by both authors using the critical appraisal skills programme CASP [9] for quality assessment. The authors then discussed their reviews collaboratively, as well as summarizing key findings from the studies which were grouped into themes, to reach agreement. To note, articles that included people with a laryngectomy and carer lived experience were meshed in this synthesis rather than being reviewed individually.

**FIGURE 2:**
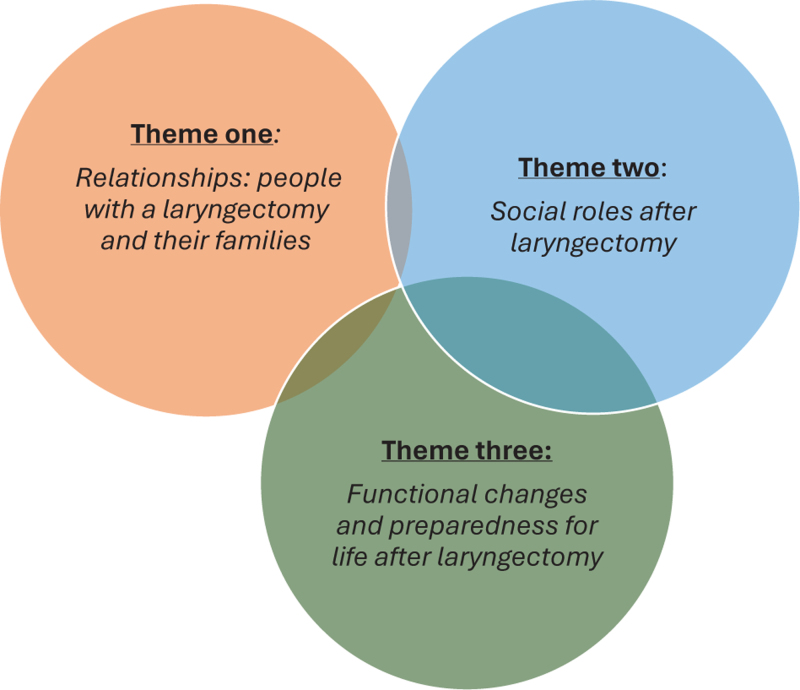
Figure representation of thematic relationships.

The review has been organized into key themes: relationships: people with a laryngectomy and their families, social roles and functioning, functional changes and preparedness for life after laryngectomy (Fig. 2).

## STUDY DEMOGRAPHICS: PARTICIPANT CHARACTERISTICS

Studies originated from the UK and Ireland [15^▪^,16^▪^], USA [14^▪▪^], Canada [17^▪^], Australia [10^▪^,^11^^▪▪^,^12^^▪▪^], Netherlands [20^▪▪^], Italy [13^▪▪^,18^▪^], China [19^▪^]. Most studies included a mix of male and female participants [10^▪^,^11^–^14^^▪▪^,^15^,16^▪^], however many participants in these studies were male, as expected within the laryngectomy population. Two studies predominantly comprised male participants [17^▪^,18^▪^], one focussed on males only [19^▪^], and one on females only [20^▪▪^]. To note, the carer participants in Dri's study were majority female [18^▪^]. In terms of age, participants ranged from 41 [13^▪▪^,^14^^▪▪^] to 90 years old [13^▪▪^]. The total number of people with a laryngectomy in the studies was 106, ranging from 8 (20) to 19 (19). For carers, the total number in the studies was 21, with a range of 9 (18)–12 (11, 12). Only three studies explicitly report on ethnicity in their participant sample [10^▪^,^11^^▪▪^,^12^^▪▪^]. Socio-economic status is stated in three studies [11^▪▪^,^12^^▪▪^,15^▪^], with the remaining studies reporting on one or two markers of socio-economic status only, for example, education, employment, income.

Participants in the studies used a range of communication methods. Participants in Bickford's [10^▪^,^11^^▪▪^,^12^^▪▪^], Gresham [14^▪▪^] and Watson's studies [15^▪^] included tracheoesophageal speech (TES), oesophageal speech (OS), electrolarynx, silent articulation and writing. Some studies only included TES users [16^▪^,17^▪^,^20^^▪▪^] or people using OS/silent articulation [13^▪▪^]. Yang's study included participants using TES or silent articulation [19^▪^]. Dri's study, focussing more on the carers experience, did not specify laryngectomy participant's communication methods [18^▪^].

Studies included participants at a range of time-points postlaryngectomy. Two papers focussed on the first 6–12 months postsurgery [17^▪^,19^▪^], one paper within the first 2 years postsurgery [15^▪^] whilst others focussed on any time-point after 1-year postsurgery [10^▪^,^11^^▪▪^,^12^^▪▪^,^14^^▪▪^,^20^^▪▪^]. Two papers focussed more broadly on time-points ranging from several months to several years postsurgery [13^▪▪^,16^▪^]. Dri's paper did not specify length of time postsurgery, or the length of the relationship that caregivers had with the person with a laryngectomy [18^▪^] (Table 1).

**Table 1:** study demographics

	Origin	Gender	Number of laryngectomy participants	Age: laryngectomy participants (years)	Number of carers	Age: carer participants (years)	Ethnicity	Socio-economic status	Communication method (*n*)	Time since laryngectomy (years)
Bickford 2013 [10^▪^]	Australia	M/F	12	57–75	0	N/A	Caucasian	1–2 markers reported	TES (9), EL/EL+OES (3)	2–11
Bickford 2018 [11^▪▪^]	Australia	M/F	12	57–75	9	36–77	Caucasian	Yes	TES (9), EL/EL+OES (3)	2–11
Bickford 2019 [12^▪▪^]	Australia	M/F	12	57–75	9	36–77	Caucasian	Yes	TES (9), EL/EL+OES (3)	2–11
Ghirotto [13^▪▪^]	Italy	M/F	19	41–90	0	N/A	Not reported	1–2 markers reported	OES (10), SA (1)	<1 – 5+
Gresham [14^▪▪^]	USA	M/F	17	41 – 80	0	N/A	Not reported	1–2 markers reported	SA (1), EL (5), OES (2), TES (9)	1–19
Watson [15^▪^]	UK & Ireland	M/F	10	<50–70+	0	N/A	Not reported	Yes	SA (not stated), EL (not stated), OES (not stated), TES (not stated)	<1 (6), >1 (4)
Noonan [16^▪^]	UK & Ireland	M/F	10	55–75	0	N/A	Not reported	1–2 markers reported	TES (10)	1–4+
Dooks [17^▪^]	Canada	M/F (mostly M)	9	60–75	0	N/A	Not reported	1–2 markers reported	TES	<1
Dri [18^▪^]	Italy	M/F (mostly F carers)	11	Not reported	12	47–76	Not reported	1–2 markers reported	Not reported	Not reported
Yang [19^▪^]	China	M	19	44–76	0	N/A	Not reported	1–2 markers reported	TES (not stated), other (not stated)	<1
Sluis [20^▪▪^]	Netherlands	F	8	60–77	0	N/A	Not reported	1–2 markers reported	TES	1–31

*n*, number; EL, electrolarynx; M/F, male/female; N/A, not applicable; OES, oesophageal speech; SA, silent articulation; TES, tracheoesophageal speech.

## THEME 1: RELATIONSHIPS: PEOPLE WITH A LARYNGECTOMY AND THEIR FAMILIES

The importance of relationships and support from family, friends and healthcare professionals in both the immediate recovery and long-term acceptance phases were central in four papers [12^▪▪^,15^▪^,17^▪^,18^▪^]. Support was critical for both the person with the laryngectomy and their caregivers:

*“There was some nice characters of the nurses … funny … make a joke with iz … just didn’t look at iz any different to any other patient which was really good … It made iz feel normal … it made iz forget” (person with laryngectomy, male)*[15^▪^].

For people with a laryngectomy, these relationships were meaningful and helped to support acceptance and adjustment to life after laryngectomy. For example, a participant in Bickford's paper talked about the support from his social circle:

*“… when I first got home from here, I went and seen them and there was no big deal … you’ve got to get on with it and that's it ” (person with laryngectomy, male)*[10^▪^].

Participants in the studies talked about the care and understanding that people provided which helped to support them through the phases of acceptance and adjustment. For some participants in the studies support from other people with a laryngectomy was important for this:

*“You know X she would say ‘are you alright Y?’ and you know I would try, and I would say ‘I’m, I’m a-a-a-alright X’ you know that's the way I was sounding. She says, ‘Y it's great to hear you flower’, and erm I really took to her well you know.” (person with laryngectomy, female)*[15^▪^].

The knowledge that healthcare professionals had around laryngectomy supported this [12^▪▪^,15^▪^], with people with a laryngectomy reporting a less positive relationship with those healthcare professionals who appeared to have less knowledge or skills. For example, a caregiver in Bickford's study reflected on her partner's reaction to a particular interaction with healthcare professionals:

“But it's like when [partner's name] had things and they try and put oxygen on there. [I] last time he said “look, you may as well put that on my a***.” (carer, female)[12^▪▪^].

Overall, the caregivers experience in the studies reported similarities to the person's living with a laryngectomy. However, there were some differences, reported in Dri's paper, specifically around the change to social interaction for the caregiver and feelings around being at home:

*“when he was in the hospital, one could call out at any time. At home instead I was always afraid.” (carer, female)*[18^▪^].

## THEME TWO: SOCIAL ROLES AFTER LARYNGECTOMY

The theme ‘social roles after laryngectomy’ was explored in six of the papers reviewed [10^▪^,^11^^▪▪^,^13^^▪▪^,17^▪^,19^▪^,^20^^▪▪^]. Regarding social roles, people talked about re-establishing meaning within their social roles whether that be within the family, with friends or with colleagues. Some people adapted the role they had within these settings due to their functional limitations e.g., changes to communication, or their altered sense of self.

Men and women reported different perceptions of loss in the literature. In general, women appeared to follow a more emotional path of adjustment [10^▪^,^13^^▪▪^,^20^^▪▪^]:

*“my life really is over … it happened overnight and you didn’ t have time to think about it … it died overnight … everything … my job, my voice, my friends, my social life. I don’ t go … anywhere! … if I do I go … like my children’ s birthdays, … I do the cooking; you don’ t have to talk to anyone … you are just part of the furniture and … meld into the background.” (person with laryngectomy, female)*[10^▪^].

Men seemed to be more practical in their approach to adjustment following laryngectomy:

*“The first thing is that I really don’t care. I move on… Of course, the thought is not that it's not there. But when I realise it, I don’t say: ‘I can’t do this’. I think of it, but I face it” (person with laryngectomy, male)*[13^▪▪^].

A further specific example of this was around adjustment and perception of communication changes; however, both men and women talked about an emotional loss when discussing their postlaryngectomy communication:

*“I am … very embarrassed … well people don’ t ask you questions because they are embarrassed to talk to you… you’ve only got to see someone’ s face … when you talk to them … and you put your hand-up and the voice comes out … They treat you differently straightaway.” (person with laryngectomy, female)*[10^▪^].

And:

*“things aren’t spontaneous anymore. … You can’t have the quick sort of stab … Particularly, if you are looking for a laugh and if you miss that split second it is gone … which of course with this (laryngectomy)… there is every likelihood that you are not going to be heard” (person with laryngectomy, male)*[10^▪^].

Despite some of these subtle, noted differences, following a period of destabilization, both men and women were similar in trying to find a positive outlook to adjust to life with a laryngectomy. However, this seemed to be somewhat more successful for men than for women. Following this destabilization period men described achieving a return to physical function [13^▪▪^,17^▪^]:

*“Getting people to accept me for what I was and what I could still do. All of sudden, because of this hole in my throat, oh, maybe I should help you do that, oh no, you shouldn’t do that anymore. Hey wait a minute, I can still pull a wrench, I can still go out and diagnose my machine I’m a maintenance manager at (a company). I’ve walked that floor, that's my floor, those are my machines. Don’t give me this stuff because I’ve got to hold my thumb to my throat to talk, that I’m any less than I was before I left this floor.” (person with laryngectomy, male)*[17^▪^].

Women described a more emotional path when returning to stability sometimes with improved realization of themselves and happiness. However, there remained a persistent vulnerability with women in adjustment to life after laryngectomy, for example, their role within their household and family:

*Taking care of the household and not wanting to delegate [tasks] […] is really very difficult for me. […] I now need a whole week for things that previously took me one day to accomplish. (person with laryngectomy, female)*[20^▪▪^].

There were also wider and important issues around intimacy to be aware of and consider, which were mainly explored in Sluis's paper [20^▪▪^], as well as Ghirotto [13^▪▪^]. For example, dating after laryngectomy, initiating conversations around intimacy with partners and potential fears around coughing and changes to communication:

*“The single participant had her total laryngectomy at the age of 45 and did not find a partner after surgery. She started to date after her rehabilitation period. Before the procedure, she experienced being quite popular with the opposite sex. After being rejected repeatedly because of her voice after the surgery, she stopped dating.” (person with laryngectomy, female)*[20^▪▪^].

*“For example, presenting myself to a woman… is no longer the same as before… It is difficult to speak, there is phlegm, there is the stoma, which is not a beautiful presence or sight to see, and consequently, it disturbs one's intimacy there too.” (person with laryngectomy, male)*[13^▪▪^].

## THEME THREE: FUNCTIONAL CHANGES AND PREPAREDNESS FOR LIFE AFTER LARYNGECTOMY

Six papers specifically explored the theme ‘functional changes and preparedness for life after laryngectomy’ [10^▪^,^11^^▪▪^,^13^^▪▪^,15^▪^,17^▪^,19^▪^]. People talked about the importance of a laryngectomy preoperative visitor and often referred to this as being more powerful than medicalized information provided. The medicalized information people received was often found to be too technical or overwhelming to navigate through [15^▪^,^20^^▪▪^] and people wanted this to be more realistic and practical.

*“Two respondents said the counselling they received was too technical: they missed receiving information about the daily implications of having a total laryngectomy.” (person with laryngectomy, female)*[20^▪▪^].

*“In some parts they explained it okay but in other parts there were some words…. bigger words I couldn’t understand so I had to ask about them.” (person with laryngectomy, female)*[15^▪^].

Functional changes discussed by participants included airway, swallow and communication, as well as body image [10^▪^,^11^^▪▪^,15^▪^,17^▪^]. People also all talked about adjustment to and acceptance of functional changes impacting on life after laryngectomy. The most discussed functional change concerned the importance of communication rehabilitation, particularly given its wider impact on an individual's overall functioning and quality of life after laryngectomy. Specific examples of this included the impact this had on relationships, participation in social settings, family role and work [10^▪^,^11^^▪▪^,^13^^▪▪^,17^▪^,^20^^▪▪^]. This finding is closely linked with theme two concerning the impact on social roles after laryngectomy.

*“First of all, I don’t work. I had to quit my job because I’m a machinist, a lot of smoke and everything else there, so I quit my job. That was very unfortunate, because I like to go to work.” (person with laryngectomy, gender not specified)*[13^▪▪^].

*“I don’t socialise like I used to … even with family… and sometimes it doesn’t affect you and other times it does affect you. It upsets you.” (person with laryngectomy, female)*[11^▪▪^].

*“And with my kids…at the shops, I would clap my hands and then they’d know: oh mama is calling. Because I didn’t want to shout them in such a shopping mall. You really don’t want to stand with your unusual voice, then.” (person with laryngectomy, female)* [20^▪▪^,^21^].

There were differences in responses to how they and other people viewed themselves after laryngectomy, mainly explored in Bickford's papers [10^▪^,^11^^▪▪^]. This may be a helpful way to think about interventions depending on an individual's sense of self – highlighting the importance of shifting away from impairment-based interventions alone.

*“During that whole time that he was talking to me. He wasn’t looking at me… I was listening to what he is saying, and he was contradicting himself because I think he thought I was stupid.” (person with laryngectomy, female)*[11^▪▪^].

*“I have still got a normal life. I do everything. I can still talk from the mobile. I have had no regrets. It had to be done. It was that or I’d be kicking up daisies.” (person with laryngectomy, male)*[11^▪▪^].

## CONCLUSION

This increased body of lived experience literature provides clinicians with a unique insight into life after laryngectomy across a variety of time-points and stages following surgery. The literature to date supports the need to shift rehabilitation care away solely from the medical model and more towards an integrated approach with a psychosocial model of care. Although missing from most of these studies, a step beyond this would be to consider a biopsychosocial model to ensure that biological factors, such as physiological processes after laryngectomy, are included alongside medical, psychological and social factors. Integration of the medical and biopsychosocial model requires further research to understand how best to achieve this within the healthcare system.

## Acknowledgements

*None.*

*Declarations: Laura-Jayne Watson is in receipt of funding from the NIHR Doctoral Clinical Academic Fellowship (DCAF) scheme. This work forms part of this fellowship.*

### Financial support and sponsorship

*National Institute of Health Research, Doctoral Clinical Academic Fellowship (NIHR 335181) (LJW).*

### Conflicts of interest

*There are no conflicts of interest.*

## REFERENCES AND RECOMMENDED READING

Papers of particular interest, published within the annual period of review, have been highlighted as:

▪ of special interest
▪▪ of outstanding interest
